# Typhoid Fever in the Military Service

**Published:** 1899-06

**Authors:** 


Monthly Review of Medicine and Surgery.

*EDITORS:*

THOMAS LOTHROP, M. D. - WM. WARREN POTTER, M. D.

All communications, whether of a literary or business nature, should be addressed to the managing editor: 284 Franklin Street, Buffalo, N. Y.

Vol. XXXVIII. JUNE, 1899. No. 11.

### TYPHOID FEVER IN THE MILITARY SERVICE.

WHILE the subject of camp typhoid has been frequently discussed of late both in medical societies and in the journals, it is by no means exhausted. This remark has all the more cogency in view of the fact that no plan has yet been devised to prevent the spread of this absolutely preventable disease in the camps of our soldiers.

We have been still more impressed with this fact since the publication of a paper by Dr. Victor C. Vaughan, of Ann Arbor, read at the recent meeting of the Association of American Physicians at Washington; and especially in reading the discussions thereon. It will be remembered that Surgeon-general Sternberg last summer appointed a commission composed of Major Walter Reed, Surgeon U. S. army and Majors E. O. Shakespeare and Victor C. Vaughan, surgeons U. S. Volunteers, to investigate the causes of typhoid fever in the army camps and report upon a method looking to the prevention of its recurrence in the future.

The paper of Dr. Vaughan above referred to is, practically, a synopsis of the work of this commission, although it is not yet completed, hence this may be regarded in a sense as a preliminary report. In the pursuit of the investigation all the large camps in the United States have been visited, personal inspections have been made, sources of water supply have been studied, quantity and quality of food looked into, nature of soils investigated, sinks, mess tents, disposition of refuse and fecal matter examined; indeed, every known factor contributing to the propagation of typhoid has been overhauled and thoroughly delved into.

Dr. Vaughan says that one of the first difficulties met was that of incorrect diagnosis, typhoid fever being most generally mistaken for protracted malarial fever. Competent experts were obtained, scientific examinations of the blood made and the Widal test applied in febrile cases. These examinations disclosed the fact that so-called malaria was a very rare disease among the soldiers in the United States, not one case having been found at Camp Alger, and but one at Chickamauga. Dr. Vaughan says there can be no doubt that reported cases of malaria in almost every instance were typhoid fever.

The infection was very general, more than 90 per cent. of the volunteer regiments becoming so before being ordered to government encampments, having brought it from home or from the state encampments. Inquiring into the methods through which the disease was spread, it was determined that the water supply of the larger camps was not infected, strong evidence of which existed in the fact that at many places the soldiers and citizens received their drinking water from the same sources and yet there was no typhoid among the citizens. How, then, did the disease spread? It was not due to sending Northern men into a Southern climate; nor yet to the locality, or to the massing of men at a given place; but, says Dr. Vaughan, it was caused by the improper disposal of excreta; that is to say, by camp pollution therewith. The paper states that the men's feet distributed the excreta; that flies carried it to food; and through this avenue was it spread.

The inevitable conclusion to reach from all this is that the only means of preventing a spread of infection is the complete sterilisation of fecal matter in camps that are to be occupied three weeks or longer.

Dr. Vaughan's paper received marked attention and was discussed by a number of the members, including Surgeon-general Sternberg, himself. Dr. Sternberg in the course of his remarks deplored the fact that the lessons of the civil war should be so soon forgotten and referred to his sanitary circular of April 25, 1898, in which he pointed out the dangers of infection and explained how it was to be avoided. He said that doctors in civil life do not pay sufficient attention to the sterilisation of the excreta of typhoid patients. These were the men who became medical officers in regiments, and it could be easily seen how neglectful they would become of this important matter. He said the trouble was the surgeons knew very little of sanitation because of their inefficient training in the medical schools, most of which pay little or no attention to sanitation or hygiene.

Dr. Jacobi remarked that he was ignorant as to the duties and powers of the surgeon-general, but he thought that officers should not be compelled to accept all the rubbish foisted upon him by an ignorant governor.

In reply Dr. Sternberg said that he did not mean to be understood that the men who went with the regiments were below the average; indeed, that they were no worse than many left at home, prevented by large practices from accepting army appointments. He further pointed out that one of the chief difficulties in the way of enforcing camp sanitation among the volunteers lay in the fact that line officers for the most part ignored the surgeon's recommendations, declaring they would pay no attention to "doctors' fads."

Dr. George Dock, of Ann Arbor, said that most volunteer surgeons started out with the expectation of fighting only malaria, and that some surgeons gave as much as thirty to forty grains of quinine a day, but did not recognise the typhoid patient that was also suffering from an overdose of quinine.

Dr. Solis-Cohen, of Philadelphia, came to the defense of the volunteer regimental surgeon, citing the record of a Pennsylvania regiment that had almost no fever, and but two deaths from any cause. This he claimed was due to the efficiency of the medical and line officers who worked together to carry out proper sanitary measures. He did not believe that surgeons were wholly responsible for the ravages of typhoid, but agreed with Dr. Sternberg in charging the responsibility home to the line officers, who, for the most part considered sanitary suggestions as medical fads.

Some other members participated in the discussion, but these were the main points developed. Dr. Vaughan in concluding remarked that the average volunteer surgeon was better than the average doctor in civil life, and insisted as the point of the whole matter that the medical officer is powerless unless the line officer upholds him and follows his directions. He suggested the propriety of teaching at West Point to the future line officers of the army a complete knowledge of sanitation and hygiene. He said the medical officers in the late war had hard work thrown upon them and that they did it well in spite of the difficulties.

It seems to us the effect of this paper and especially its discussion ought to prove beneficial. Much of it follows lines mapped out by the Buffalo Sanatory Club in its work last winter, and by the Medical Society of the State of New York in its discussion of a similar problem.

It would seem that these several intelligent discussions of camp sanitation and the danger of infection by its neglect, ought to stimulate congress to arm the Surgeon-general with ample powers to prevent a repetition of the direful calamity that befel us in the Spanish war.

---

ST. CLAIR MCKELWAY, ESQ., editor of the *Brooklyn Eagle* and regent of the University of the State of New York, made a speech at the annual banquet of the Associated Press, which was held at the Auditorium Hotel, Chicago, May 17, 1899.

In the course of his speech, the theme of which was Press and the Times, Regent McKelway, who was received with much enthusiasm said :

This war found an indissoluble Union of indestructible states, compact of purpose for liberty and humanity. Other interests may tell their own story of participation in the duties and of right in the honors of that conflict. Our own calling has no story to tell of that kind. For the telling of the story of the war itself was our participation in it, and the right and the true telling of it is our claim to a share in its honors. By universal admission, that has been well done. There was unreadiness in our offices for the work. But so there was in the War Department. There was an inadequacy of staff organisation, so to speak, in our offices for the duties devolving on us. But so there was in the same department. And in our cases that inadequacy was repaired. New functions and new forces were extemporised—and not found wanting. New blood was sought and found. New methods were devised. And no commission of investigation and no court of inquiry have been ordered to sit upon our derelictions, to pass on our incidental errors, or to sift our mistakes of the past for the greater security and sufficiency of the future.

I wish in all seriousness, to say that the press of the United States, has, in the main, well served the country. A percentage of our newspapers, as large as ninety-five out of every hundred, sustained the war. The few which did not sustain it in part atoned for that by fairly and fully reporting it. They exhibited the not undesirable exceptions which illustrated the general excellence of our common calling.

It must be admitted that the unity of the press, the substantial unity of the press, for the war has not been followed by absolute unity among its members on the results of the war. Fixed facts, however, have commanded that respect, even with newspapers, which they generally command everywhere. There is no newspaper which believes that we are in Porto Rico ever to get out. We are there to stay. There is none which believes that we are in Cuba to get out—soon. I think we will stay there about as long as Great Britain will stay in Egypt, and that Great Britain will stay in Egypt about as long as the Anglo-Saxon race has a habit of staying where it settles down. The duration of our stay in the Philippines is prodigiously debated. While the debate goes on we stay. If the debate coincides with our stay, I think it will be a protracted debate.

Let us, as newspaper men, bear a few facts in mind, whether we like them or not. One is that the Constitution is the Constitution of the States and not of the Territories, except as it may be extended over the territories by legislation. Another is that territories unorganised by congress are under the powers which this nation has, as a nation, and which it has without regard to its Constitution. These powers are absolute. This nation had them when it declared its independence and before it made its constitution. In those powers are involved the right of existence, of defence, of aggression and of conquest. The Constitution divided up certain powers among the states and in the general government, but it neither made our nationality nor limits our national powers in dealing with other nations. The territories which we acquire may come within the rights of the Constitution in the ways by law provided, but they can come in no other way, and until they do come in or are brought in they may have rights, but they are not constitutional rights. They may suffer wrongs, but they will not be constitutional wrongs; they are under the control of our nation, as a nation, and of our government as the representative of a nation in its national as well as in its more strictly defined constitutional powers.

Another thing to bear in mind is that we could not leave Spain in the Philippines and could not restore the Philippines to Spain without making ourselves worse than Spain in history, and without putting or leaving a national enemy to work us harm in the very heart of the commercial world of the far East. I need not talk to those that think that the Filipinos are capable of self-government. Men who can believe that can believe anything. The Filipinos comprise many tribes at odds with and strangers to one another, differing in religion, in language and in habits, and some of them have been left for centuries in the savagery of which head-hunting and cannibalism are perhaps the mildest variations. The more intelligent of them, who have unwisely confronted our troops, are led by men who will be without occupation when they have no cause to sell and no government to market. Those who can regard them as heroes, patriots, as virtuous, and as intelligent, resembles the lecturer whose boast it was that he made his facts as he went along. All the evidence is against them.

I take it, we are going to hold those islands. I doubt not we shall find difficulty. Difficulties, however, alter not duties. No man living knows what we will do with them or should do with them, for we are learning as we go along. The situation was not of our making, could not be of our preventing, and will not be one which we will abandon or run away from. The Philippines are a finality. We have them and we hold them. We will govern them under the universal right and obligation of the capable to govern the incapable, cast upon their hands. We will do them the good which the Pilgrim and the Quaker, the Huguenot and the Cavalier, would have done to the Indians, had the Indians had sense enough to accept it. They had not, and the rest followed. Good, or the consequences of the rejection of it, will follow in the Philippines. The world will not stop; it will go right on. The higher races will school or harness the lower ones to the work of the ages, and American newspapers should not captiously forget that fact in their long outlook on events.

